# The impact of mergers and acquisitions on technological innovation in state-owned enterprises: The moderating role of mixed-ownership

**DOI:** 10.1371/journal.pone.0324025

**Published:** 2025-05-28

**Authors:** Niannian Wu, Furong Guo, Bingxia Wang

**Affiliations:** 1 School of Accounting, Chongqing Technology and Business University, Chongqing, China; 2 Institute of Mathematical Sciences, Faculty of Science, University of Malaya, Kuala Lumpur, Malaysia; Sadat Academy for Management Sciences, EGYPT

## Abstract

Using data from Chinese A-share listed state-owned enterprises (SOEs) between 2007 and 2019, we examine how mergers and acquisitions (M&As) affect SOE innovation through patent outputs, with a focus on mixed-ownership M&As where SOEs acquire private firms. Our results show that while M&As generally enhance SOE innovation through increased patent applications, mixed-ownership M&As demonstrate significantly stronger positive effects compared to SOE-to-SOE M&As. This enhancement is most notable when control rights are transferred, when acquiring SOEs possess high R&D investment but lower production efficiency, and in regions with less developed markets. The primary mechanism appears to be improved corporate governance through increased private shareholder involvement in strategic decision-making. These findings advance our understanding of how ownership differences influence innovation in M&As while providing practical guidance for SOE reform policies in China and similar emerging economies.

## 1. Introduction

Enterprises play a crucial role as drivers of innovation and serve as vital links between scientific advancement and economic growth. State-owned enterprises (SOEs) have emerged as significant players across various economies, with mergers and acquisitions (M&As) becoming an increasingly important strategy for their development (Reddy et al., 2016). While early research raised concerns about the effectiveness of SOE M&A decisions [1,2], this study examines how M&As affect technological innovation in SOEs, with particular attention to the ownership type of target firms.

The relationship between M&As, target firm ownership, and technological innovation in SOEs warrants careful investigation. Mixed ownership, which combines state and private capital, has shown promise in addressing traditional corporate governance challenges [3]. Through capital markets, SOEs can both attract private investment and acquire private enterprises [4], potentially enhancing both their market position and operational quality.

Our research suggests that M&As, especially those involving private firms as targets, can enhance SOEs’ innovative capabilities. Our findings challenge previous research that emphasized inefficient governmental intervention in M&A decisions [5–7]. While contemporary SOEs increasingly prioritize operational efficiency and market competitiveness [8], they still maintain unique advantages in accessing social capital and innovation financing through institutional connections [9]. Meanwhile, private firms bring complementary strengths in market-oriented governance and investment monitoring [10,11]. Our empirical analysis demonstrates that this combination of resources through mixed-ownership M&As creates stronger innovation outputs compared to M&As between SOEs.

We examine China’s A-share listed SOEs for several compelling reasons. China’s institutional environment creates distinct resource allocation patterns and policy support mechanisms across ownership types [12]. The country’s ongoing mixed-ownership reforms aim to enhance SOEs’ market orientation while maintaining their strategic importance [13,14]. While this study focuses on China’s context, its findings offer valuable insights for other emerging markets facing similar innovation and governance challenges. These lessons are particularly relevant in areas such as institutional adaptation, innovation capability enhancement, and market-oriented reforms.

To address methodological challenges in identifying target firm ownership, we carefully examined M&A announcements, annual reports, and related documentation. Based on manually collected M&A data from Chinese A-share listed SOEs (2007–2019), we employed propensity score matching and difference-in-differences (PSM-DID) models to address endogeneity concerns. Our baseline results indicate that M&As significantly increase SOEs’ patent applications, with mixed-ownership transactions demonstrating particularly strong effects.

We also find that the positive impact of mixed ownership is more pronounced in transactions involving control rights transfer, SOEs with good innovation resources but low efficiency, and regions with lower market development levels. This suggests that mixed-ownership M&As are particularly effective in situations where control rights transfer enable more substantial governance reforms, where there is greater potential for efficiency improvements, and where institutional barriers to innovation are more significant.

To understand the underlying mechanisms, we examined changes in corporate governance following these M&As. Our analysis shows that mixed-ownership M&As lead to increased participation of non-state shareholders in SOE governance, which appears to be a key driver of the innovation advantages observed in mixed-ownership arrangements. These findings suggest that M&As, particularly those involving mixed ownership, effectively address historical limitations in SOE governance and successfully unlock innovation potential.

This research contributes to existing literature in three significant ways. First, it extends the M&A and innovation literature by specifically examining mixed-ownership M&As as a distinct type, addressing a research gap in understanding how ownership differences influence innovation outputs. Second, it advances mixed-ownership reform literature by providing empirical evidence on how SOEs’ equity participation in private enterprises affects their own technological innovation capabilities, offering a more comprehensive perspective on the economic consequences of mixed-ownership reform. Third, our study not only demonstrates the positive impact of mixed-ownership M&As on SOE innovation but also empirically examines the underlying mechanisms through which M&A type heterogeneity affects non-state shareholder participation. By investigating the roles of transaction features, firm characteristics and external environment, we provide deeper insights for both theoretical development and practical implementation.

The remainder of this paper is organized as follows. Section 2 reviews the literature. Section 3 presents our theoretical analysis and develops hypotheses. Section 4 details our research design. Section 5 presents empirical results and robustness analysis. Finally, Section 6 concludes with a discussion of our findings.

## 2. Literature review

### 2.1. M&As and innovation

The relationship between M&As and innovation has long been a significant topic in academic research [15,16]. While existing research has extensively examined general M&As, the role of ownership differences in M&A-innovation relationships remains understudied, particularly in emerging economies where state ownership plays a crucial role.

Drawing from recent developments in resource dependency theory, scholars identify the acquisition of knowledge and intangible resources as central to realizing synergistic value in M&As [17–19]. M&As enhance innovation through four key pathways: scale effects, accelerated market entry, technology acquisition, and innovation synergies [20–22]. However, empirical studies show mixed results: while some research finds that M&As significantly improve innovation performance [23–25], others point to potential negative effects [26–28]. These differences mainly stem from factors such as organizational complementarity and cultural fit [29,30].

Despite extensive research on various aspects of M&As’ impact on innovation, the specific dynamics of mixed-ownership M&As remain unexplored. This gap is particularly notable in emerging economies, where the interaction between state and private ownership may create unique implications for post-M&A innovation outputs. While existing research has focused primarily on general M&As or private investment in SOEs, the innovation effects of SOE investment in private enterprises represent an important yet understudied phenomenon.

Open innovation theory provides a new framework for understanding this complexity [31,32]. Within this framework, M&As serve not only as a means of acquiring external knowledge but also as a crucial mechanism for promoting knowledge flow and expanding innovation networks [33–35]. This theoretical perspective is particularly relevant for understanding SOE M&As and innovation, as it emphasizes how institutional environments influence innovation outputs.

### 2.2. State-owned enterprise innovation and mixed-ownership reform

SOEs worldwide face persistent challenges in innovation efficiency, as documented by extensive research [36,37]. Previous studies have consistently found that these challenges stem primarily from excessive government control, weak incentive systems, and low innovation drive [38,39]. This apparent innovation inefficiency in SOEs has led to various reform attempts globally, with mixed-ownership reform emerging as a potential solution.

Countries have adopted diverse approaches to mixed-ownership reform, reflecting different institutional contexts and reform objectives. While developed economies like the UK, France, Germany, and the US have emphasized market mechanisms to varying degrees [40,41], emerging economies face distinct challenges in their reform processes. China’s experience since 1997, particularly after the 2013 ownership diversification reforms, offers unique insights [42,43]. Studies have found that introducing non-state shareholders to Chinese SOEs has led to improved innovation outputs through enhanced governance mechanisms, market-oriented incentives, and reduced government interference [44–47]). However, these improvements show significant variation across ownership structures and industries [48]. In contrast, other emerging economies present different reform trajectories: Vietnam’s gradual approach, India’s aggressive privatization, and Brazil’s politically constrained reforms [49–53].

Despite these reform efforts, significant knowledge gaps remain in understanding mixed-ownership’s impact on innovation. While existing research has extensively examined private investment in SOEs [44,46,54], studies on state investment in private firms remain limited in scope [55], focusing primarily on changes within acquired companies [56,57]. This narrow focus reflects our incomplete understanding of mixed-ownership reform’s complexity [13,58].

Our literature review reveals three significant research gaps. First, while M&As are known to drive technological innovation, their effectiveness in SOE reforms needs more study. Second, while research has focused on private investment in SOEs, it has overlooked the impact of SOEs investing in private firms. Third, we lack understanding of how mixed-ownership affects technological innovation when SOEs acquire private firms, especially in developing economies. Our study addresses these gaps by examining how SOE M&As impact technological innovation and how mixed-ownership moderates this relationship. This research contributes to corporate governance literature, particularly in public-private cooperation [59,60].

## 3. Theoretical analysis and hypothesis development

### 3.1. How M&As impact innovation in state-owned enterprises

State-owned enterprises (SOEs) play a crucial role in national development. Their innovation capabilities are shaped by ownership structure [43]. This section analyzes M&As’ impact on SOE innovation through three theoretical lenses.

From a resource dependence perspective [61], SOEs rely heavily on external resources. Their political connections provide privileged access to technological fields, national science projects, and research partnerships [62]. However, this government resource dependence can constrain innovation flexibility [63,64]. Through the resource-based view [65], SOEs possess unique advantages in financing capabilities and R&D investments [66]. Yet they often lack the market responsiveness and knowledge absorption capabilities found in private enterprises [10]. Agency theory [67]highlights how multiple principal-agent relationships in SOEs create complexity in innovation decision-making. This complexity affects the efficiency of resource allocation and strategic choices in innovation processes.

M&As function as an open innovation pathway by enabling SOEs to access and integrate diverse knowledge sources [68]. Through M&As, SOEs can obtain new technologies [34], enhance their innovation capabilities, and build innovation ecosystems [32,69]. This integration of external capabilities with SOEs’ existing resources helps overcome traditional innovation barriers.

Drawing from these three theoretical perspectives - which highlight resource access, capability development, and governance challenges - we propose:

H1: M&As enhance SOEs’ technological innovation capabilities.

### 3.2. Mixed-ownership M&As and innovation

Drawing on institutional theory, resource dependence theory, and agency theory, we analyze how mixed-ownership M&As affect innovation capabilities through complementary theoretical lenses. Institutional theory suggests that different ownership structures embody distinct institutional logics and governance mechanisms [70]. Resource dependence theory emphasizes how organizations seek external resources through strategic alliances to enhance their competitive advantages [71]. Agency theory provides insights into how ownership structure affects monitoring and incentive mechanisms that influence innovation decisions [67].

These theoretical frameworks help explain the fundamental differences between SOEs and private enterprises in innovation. SOEs possess institutional advantages in accessing national science projects, research partnerships, and financing capabilities [62,66]. Private enterprises, operating under market-oriented institutional pressures, excel in profit-driven innovation, rapid market response, and knowledge absorption [10,11].

Based on these theoretical perspectives, mixed-ownership M&As promote innovation through multiple interconnected mechanisms. From a resource dependence perspective, complementarity emerges as SOEs contribute their institutional advantages in accessing national projects and financing, while private enterprises bring market-oriented capabilities. From an agency theory perspective, the governance structure evolves to incorporate market-based monitoring and incentive mechanisms while maintaining state support. From an institutional theory perspective, this integration helps align state and market institutional logics. When SOEs acquire private enterprises, these mechanisms work together through knowledge integration, where SOEs’ technological resources combine with private firms’ market expertise to create unique innovation synergies [29,72]. Therefore:

H2: Mixed-ownership M&As generate stronger innovation effects than M&As between SOEs.

### 3.3. Mixed-ownership M&As and non-state shareholder participation in governance

Mixed-ownership M&As combine state and private ownership, creating opportunities while presenting coordination challenges between different ownership approaches [73], particularly in aligning different institutional logics and operational methods.

The integrating of non-state capital naturally requires non-state shareholders’ governance expertise. These shareholders bring valuable market-oriented experience and integration knowledge, making their participation crucial for post-M&A success. From an agency theory perspective, their involvement optimizes monitoring and reduces principal-agent conflicts [47,67] through: (1) market-based performance evaluation systems that align management incentives with innovation; (2) enhanced board monitoring of R&D investment allocation and innovation project decisions; and (3) improved decision-making transparency that reduces information asymmetry between different stakeholder groups. Research confirms that non-state shareholders improve innovation through effective monitoring and strategic guidance [14,43,57,74].

Non-state shareholders’ active governance participation combines their specialized market knowledge with state support advantages [43]. Their market-based operational expertise particularly benefits technological innovation and entrepreneurship initiatives. Therefore:

H3: Mixed-ownership M&As enhance SOEs’ innovation capabilities by facilitating greater non-state shareholder participation in corporate governance.

## 4. Research design

### 4.1. Sample selection and data sources

This study examines equity M&As by Chinese A-share listed SOEs from 2007 to 2019. The sample period starts from 2007 because: (1) Chinese listed companies adopted new accounting standards aligned with International Financial Reporting Standards (IFRS) in January 2007, ensuring data consistency; (2) the State Council’s late-2006 guidelines on SOE restructuring guidelines marked a new reform phase. The study ends in 2019 to avoid COVID-19’s impact on M&A markets. This 13-year timeframe captures both sufficient M&A samples and complete innovation cycles.

We collected data from multiple sources (Table 1): target companies’ ownership information (SOE or private) from merger announcements, annual reports, and news releases; M&A event data and company financial characteristics from the China Research Data Services (CNRDS) database; and patent data from the China National Intellectual Property Administration (CNIPA) database, accessed through CNRDS.

**Table 1:** Main variable definitions and data sources.

Variables	Definitions	Data Sources
*Patent apply*	Natural logarithm of one plus the total number of annual patent applications	CNIPA Patent Database
*Patent_inv apply*	Natural logarithm of one plus the total number of annual invention patent applications	CNIPA Patent Database
*M&A*	The binary variable is equal to 1 if an M&A event occurs during the sample period and 0 if otherwise	CNRDS M&A Database
*Mixed-ownership*	The binary variable is equal to 1 when the target firm is private, and 0 otherwise	Manual collection from announcements, annual reports and news
*Post*	Binary variable equal to 1 in and after the year the M&A event occurs and 0 before it	CNRDS M&A Database
*Non-state governance*	The ratio of directors, executives, and supervisors appointed by non-state shareholders to total board members and executives	CNRDS Corporate Governance Database
*Size*	Natural logarithm of ending total assets	CNRDS Financial Database
*Age*	Natural logarithm of years from firm establishment date to the observation year	CNRDS Company Information Database
*Leverage*	The ratio of total debt to total assets	CNRDS Financial Database
*Capex*	Capital expenditure by total assets	CNRDS Financial Database
*Cash*	Natural logarithm of the sum of cash and cash equivalent	CNRDS Financial Database
*ROA*	Net income by total assets	CNRDS Financial Database
*MTB*	Total owner’s equity by ending market capitalization	CNRDS Financial Database
*Top1*	Percentage of ownership of the largest shareholder	CNRDS Ownership Database
*Patent_age*	Natural logarithm of average age of firm’s valid patents	CNIPA Patent Database

*Table notes* This table presents the definitions and data sources of main variables used in this study. Patent data are from the China National Intellectual Property Administration (CNIPA) Patent Database. Target firm ownership information is manually collected from M&A announcements, annual reports and news. Financial and governance data are from corresponding sub-databases of China Research Data Services Platform (CNRDS).

Following standard M&A research practices, we applied screening criteria: treating multiple acquisitions of the same target as one event; excluding financial/real estate sectors, transaction below RMB 5 million, and those under common control. We retained only each SOE’s first acquisition and excluded those with additional acquisitions within three years to isolate individual M&A effects. After removing observations with missing variables and winsorizing continuous variables at 1%, our final dataset contains 9,035 firm-year observations from SOEs, including 494 transactions (331 SOE-to-SOE and 163 mixed-ownership M&As), reflecting China’s gradual mixed-ownership reform progress.

### 4.2. Empirical model and main variables

#### 4.2.1. Model construction.

We employ a Propensity Score Matching-Difference in Differences (PSM-DID) method to address endogeneity concerns. This dual approach first matches similar firms for comparison, then analyzes acquisition effects by comparing changes before and after mergers. PSM-DID effectively addresses selection bias and omitted variable issues in M&A research [72,75], providing reliable causal inference by balancing observable characteristics and controlling for unobserved heterogeneity.

Following Moser and Voena [76], we established baseline Eq (1):


$$\begin{array}{c} \begin{array}{cc} Innovation_{i,t}=\alpha_{0}+\beta_{1}M\;\;\;\;\; & {\&A}_{i}\times Post_{i,t}+\gamma X_{i,t}+\mu_{i}+\lambda_{t}+\varepsilon_{i,t} \end{array} \end{array}$$ (1)

To examine how target firm ownership influences M&A innovation effect, we introduced *Mixed-ownership* for triple-difference analysis in Eq (2):


$$\begin{array}{c} \begin{array}{ccc} Innovation_{i,t}=\alpha_{0}+\beta_{1}M\;\;\;\;\; & {\&A}_{i}\times Post_{i,t}+\beta_{2}Mixed\text{- }{ownership}_{i}\times M\;\;\;\;\; & {\&A}_{i}\times Post_{i,t}\#\\ +\gamma X_{i,t}+\mu_{i}+\lambda_{t}+\varepsilon_{i,t} \end{array} \end{array}$$ (2)

where $Innovation_{i,t}$ represents firm*i*’s innovation outputs at time *t*; $Post_{i,t}$ indicates post-acquisition periods (1) or no*t* (0); $M\&A_{i}$ denotes treatment group (1 for acquiring firms, 0 for control); $\begin{array}{c} Mixed-{ownership}_{i} \end{array}$ represents target firm ownership (1 for private, 0 for state-owned); $X_{i,t}$ includes control variables; $\mu_{i}$ and $\lambda_{t}$ captures firm and year fixed effects respectively; and $\varepsilon_{i,t}$ is the error term.

For our baseline analysis, we use truncated OLS regression due to frequent zero patent observations, focusing on firms with existing patent applications. We ensure result reliability through additional model specifications and clustered robust standard errors, with year and firm fixed effects controls.

#### 4.2.2. Variable design and definition.

We measure corporate innovation outputs using two patent indicators: the natural logarithm of total annual patent applications (*Patent apply*) to measure overall innovation intensity, and the natural logarithm of invention patent applications (*Patent_inv apply*) to reflect high-quality innovation outputs [77,78]. To enhance the robustness of our results, we also use the number of granted patents as an alternative innovation outputs indicator [79,80]. We chose patent indicators over R&D investment as our primary measure because post-acquisition firms may consolidate overlapping R&D projects [81], while patent data provides a more objective reflection of actual changes in innovation capability [82].

Drawing from innovation economics theory and empirical research, we construct a comprehensive control framework particularly relevant to China’s mixed-ownership reform context. Our control variables encompass three key dimensions that potentially influence innovation outputs in SOEs:

First, we control for fundamental firm characteristics including size (*Size*) and age (*Age*). In the context of Chinese SOEs, these variables not only affect R&D investment capacity and innovation flexibility [83,84], but also reflect firms’ reform progress and institutional connections.

Second, we include financial indicators comprising return on assets (*ROA*), leverage ratio (*Leverage*), capital expenditure (*Capex*), and cash holdings (*Cash*). These metrics are crucial as they capture both SOEs’ market-oriented innovation investment capacity and policy-mandated resource allocation patterns [85,86].

Third, we control for governance factors including investment opportunities (*MTB*), ownership concentration (*Top1*), and innovation accumulation (*Patent_age*). These variables are particularly important in China’s mixed-ownership reform context, as they reflect the influence of ownership structure and governance mechanisms on innovation decisions [87,88]. The ownership concentration measure specifically addresses the varying degrees of ownership diversification in Chinese SOEs, which could affect both M&A decisions and subsequent innovation outputs. Table 1 lists all variables used in this study, including their definitions, measurement methods, and data sources.

## 5. Empirical results

### 5.1. Descriptive statistics

Before conducting the empirical analysis, we first present comprehensive descriptive statistics of our sample data (Table 2). Our sample data reveals that 61.8% SOEs completed M&As during the study period, with 20.4% specifically acquiring private enterprises.

**Table 2:** Descriptive statistics of key variables (2007–2019).

Variable	N	Mean	Median	Std. Dev.	Min	P25	P75	Max
*Patent apply*	9035	1.962	1.881	0.000	0.000	1.792	3.367	6.850
*Patent_inv apply*	9035	1.398	1.600	0.000	0.000	0.693	2.485	6.080
*M&A*	9035	0.618	0.486	0.000	0.000	1.000	1.000	1.000
*Mixed-ownership*	9035	0.204	0.403	0.000	0.000	0.000	0.000	1.000
*Non-state governance*	9035	0.018	0.053	0.000	0.000	0.000	0.000	0.320
*Size*	9035	6.268	1.414	3.391	5.281	6.099	7.131	10.29
*Age*	9035	2.880	0.322	1.966	2.682	2.914	3.107	3.552
*Leverage*	9035	0.514	0.212	0.069	0.360	0.521	0.668	1.064
*Capex*	9035	0.048	0.048	0.000	0.013	0.033	0.068	0.235
*Cash*	9035	0.147	0.114	0.006	0.066	0.116	0.193	0.566
*ROA*	9035	0.046	0.069	-0.279	0.024	0.045	0.074	0.240
*MTB*	9035	0.651	0.255	0.111	0.455	0.658	0.856	1.167
*Top1*	9035	0.376	0.157	0.100	0.247	0.363	0.497	0.770
*Patent age*	9035	1.317	0.742	0.000	0.962	1.459	1.825	2.773

*Table notes* This table reports the descriptive statistics of key variables in our sample. The sample consists of 9,035 firm-year observations from Chinese state-owned enterprises during 2007–2019. All continuous variables are winsorized at the 1% level. Variables are defined in Table 1. N represents the number of observations. Mean, Median, Std. Dev. (standard deviation), Min (minimum), P25 (25th percentile), P75 (75th percentile), and Max (maximum) are reported for each variable.

The sample firms show strong representatives in industry distribution, with concentrations in wholesale and retail (8.06%), energy supply (6.07%), and chemical industries (5.26%), aligning with the typical industrial layout of Chinese SOEs.

Beyond industry distribution, financial conditions are crucial sample characteristics. Financial indicators demonstrate stable performance, with an average debt-to-asset ratio of 51.4% and return on assets of 4.6%. The sample exhibits notable variations in both innovation activities and company size.

In terms of innovation output, sample firms average 45.3 patent applications annually (logarithmically transformed to 1.926 to address data dispersion), with invention patents comprising 43%. The patent application distribution shows a right-skewed pattern, consistent with established innovation research findings.

### 5.2. Propensity score matching results

To accurately assess the impact of M&As on state-owned enterprises’ innovation, we employed a Propensity Score Matching-Difference in Differences (PSM-DID) approach. This method matches state-owned enterprises that underwent M&As (treatment group) with similar SOEs that did not (control group), allowing for more precise evaluation of M&A effects.

We utilized 1:1 nearest neighbor matching technique, pairing each acquiring SOE with the most similar non-acquiring enterprise. To ensure robustness, we also employed alternative methods including nearest neighbor matching, radius matching, and kernel matching (detailed results available upon request). The matching process considered all relevant characteristics from the year before M&A and ensured paired firms were from the same industry. Industry matching is particularly crucial in China’s institutional context, where SOE M&A decisions are often influenced by policy directives and local government intervention, and there are significant variations in policy environment and market development across different sectors.

Table 3 compares the characteristics between treatment and control groups. The results show no significant differences between the groups for any variables (all p-values > 0.1). For example, the mean patent applications are 2.446 and 2.431 for treatment and control groups respectively, with a minimal difference of 0.015 (p = 0.911). Similarly, firm size means are 6.427 and 6.468, with a difference of -0.041 (p = 0.691), while firm age means are 2.913 and 2.904, with a difference of 0.009 (p = 0.682).

**Table 3:** Balance test results for PSM-matched samples.

Variable	Mean		Diff	P-value	Variable	Mean		Diff	P-value
	Treated	Control			Treated	Control			
*Patent apply*	2.446	2.431	0.015	0.911	*Patent_inv apply*	1.720	1.675	0.045	0.676
*Size*	6.427	6.468	-0.041	0.691	*Size*	6.427	6.367	0.06	0.553
*Age*	2.913	2.904	0.009	0.682	*Age*	2.913	2.920	-0.007	0.729
*Leverage*	0.511	0.506	0.005	0.726	*Leverage*	0.511	0.505	0.006	0.668
*Capex*	0.048	0.051	-0.003	0.319	*Capex*	0.048	0.051	-0.003	0.425
*Cash*	0.139	0.142	-0.003	0.695	*Cash*	0.139	0.147	-0.008	0.344
*ROA*	0.048	0.055	-0.007	0.159	*ROA*	0.048	0.049	-0.001	0.808
*MTB*	0.669	0.660	0.009	0.624	*MTB*	0.668	0.653	0.015	0.363
*Top1*	0.386	0.389	-0.003	0.776	*Top1*	0.386	0.384	0.002	0.825
*Patent_age*	1.372	1.357	0.015	0.742	*Patent_age*	1.372	1.346	0.026	0.580

*Table notes* This table presents balance test results for propensity score matched (PSM) samples comparing mixed-ownership M&As (treated group) with SOE-to-SOE M&As (control group). The left panel shows the comparison for total patent applications, while the right panel shows results for invention patent applications. Mean values are reported for both treated and control groups, along with their differences (Diff). P-values correspond to t-statistics. All variables show insignificant differences (p > 0.1) between treated and control groups, demonstrating successful matching.

**Fig 1:**
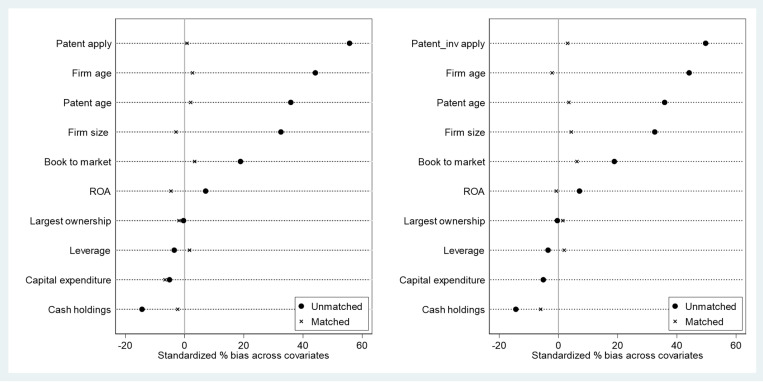
Covariate balance before and after propensity score matching. This figure presents standardized differences in covariates between treatment and control groups before and after propensity score matching. The horizontal axis shows the standardized differences, and the vertical axis lists the covariates. Smaller post-matching differences indicate better balance between groups.

Fig 1 visually demonstrates these matching results. The variables are divided into two groups by dependent variable, with PSM covariates on the y-axis and the standardized differences between control and treatment groups before and after matching on the x-axis. The results show a clear reduction in standardized differences after PSM, indicating good matching quality.

### 5.3. Baseline regression results

Table 4 presents the baseline regression results examining M&A impacts and target ownership effects on SOE innovation. Our model shows strong explanatory power with adjusted R^2^ values of 0.56–0.57. Notably, some traditional control variables like *Leverage* and *Top1* show insignificant coefficients, likely reflecting Chinese SOEs’ unique characteristics under their dual management system combining administrative and market-based approaches.

**Table 4:** Impact of M&A and target ownership on SOE technological innovation.

	(1)	(2)	(3)	(4)
	*Patent apply*	*Patent_inv apply*	*Patent apply*	*Patent_inv apply*
***M&A× Post***	0.313***	0.323***	0.205**	0.245***
	**(0.073)**	**(0.074)**	(0.085)	(0.081)
***Mixed-ownership × M&A × Post***			0.313***	0.213*
			**(0.111)**	**(0.125)**
*Size*	0.437***	0.434***	0.435***	0.435***
	(0.049)	(0.047)	(0.049)	(0.047)
*Age*	-0.558***	-0.701***	-0.565***	-0.702***
	(0.187)	(0.177)	(0.186)	(0.175)
*Leverage*	0.076	0.103	0.090	0.103
	(0.219)	(0.206)	(0.219)	(0.206)
*Capex*	-1.771***	-2.419***	-1.793***	-2.397***
	(0.558)	(0.509)	(0.559)	(0.507)
*Cash*	0.832**	0.935***	0.829**	0.930***
	(0.350)	(0.335)	(0.349)	(0.336)
*ROA*	0.275	-1.082***	0.335	-1.054**
	(0.389)	(0.416)	(0.386)	(0.415)
*MTB*	-0.572***	-0.707***	-0.559***	-0.703***
	(0.200)	(0.182)	(0.199)	(0.181)
*Top1*	0.123	-0.230	0.125	-0.240
	(0.333)	(0.274)	(0.331)	(0.273)
*Patent age*	0.123	0.015	0.123	0.017
	(0.078)	(0.072)	(0.078)	(0.072)
Constant	1.819***	2.106***	1.838***	2.103***
	(0.626)	(0.623)	(0.620)	(0.618)
Firm and year-fixed effects	Yes	Yes	Yes	Yes
Observations	6,951	6,051	6,951	6,051
Adjusted R^2^	0.555	0.573	0.557	0.574

*Table notes* This table presents regression results for the impact of M&A and target ownership type on SOEs’ innovation. Columns (1) and (2) report the baseline effects of M&As on total patent applications and invention patent applications, respectively. Columns (3) and (4) examine the differential effects between mixed-ownership M&As and SOE-to-SOE M&As. The analysis uses truncated OLS and PSM-DID methods. Robust standard errors, clustered at the firm level, are shown in parentheses. Significance levels: *** p < 0.01, ***p < 0.05, * p < 0.1.

Table 4 reveals two significant findings. First, M&As significantly boost SOEs innovation, with patent applications increasing substantially post-M&A (*M&A* × *Post* coefficients: 0.313 and 0.323, p < 0.01). This strongly supports Hypothesis 1, confirming M&As’ positive innovation impact.

Second, target firm ownership significantly shapes innovation outcomes. SOEs acquiring private enterprises show markedly stronger innovation improvements (*Mixed-ownership* × *M&A* × *Post* coefficients: 0.313, p < 0.01; 0.213, p < 0.10). This superior performance stems from private firms’ more flexible management and market-oriented innovation approaches being successfully integrated into SOEs, supporting Hypothesis 2.

These findings carry important policy implications. They validate M&As as an effective innovation enhancement tool for SOEs, meriting policymakers and manager attention. Crucially, they highlight target selection’s importance: acquiring innovative private enterprises enables SOEs to more effectively gain new technologies, knowledge, and innovation approaches, leading to transformative capabilities improvements.

### 5.4. Robustness analysis

#### 5.4.1. Parallel trends test.

To enhance the robustness of our findings and address potential endogeneity concerns, we conducted a temporal analysis of SOEs’ innovation performance during the period [*T*_-3_, *T*_+9_] surrounding M&A events [89]:


$$\begin{array}{c} \begin{array}{c} Innovation_{i,t}=\alpha_{0}+\sum_{x=-3}^{9}\beta_{x}M\&A_{i}\times T_{x}+\lambda_{t}+\mu_{i}+\varepsilon_{i,t} \end{array} \end{array}$$ (3)

In Eq (3), *x* denotes the year relative to the M&A event, $\mu_{i}$ captures firm fixed effects, $\lambda_{t}$ represents year fixed effects (controlling for macroeconomic trends, and $\varepsilon_{i,t}$ is the error term. This time window was chosen to provide sufficient data points before and after M&As for comprehensive trend analysis. To address multicollinearity concerns, we used the year prior to M&A as the cointegration baseline to distinguish, and include the interaction terms as explanatory variables in the regression. The coefficients of $M\&A_{i}\times T_{x}$ reflect of the differences between treatment and control groups in specific years.

**Fig 2:**
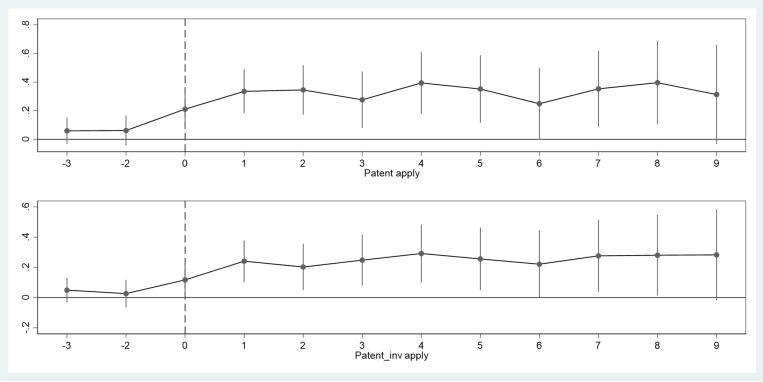
Dynamic effects of M&As on innovation outputs. This figure plots the estimated coefficients and 95% confidence intervals from the dynamic effects analysis of Eq (3), examining innovation outputs trends from three years before to nine years after M&As. The vertical axis represents the magnitude of effects on patent applications, and the horizontal axis shows the years relative to M&A completion, where year 0 represents the year of M&A completion. The insignificant coefficients in pre-M&A periods (confidence intervals containing zero) support the parallel trends assumption.

Table 5 shows that the coefficients for pre-M&A periods *T*_-3_ and *T*_-2_ are close to zero and statistically insignificant (corresponding to 95% confidence intervals containing zero in Fig 2), indicating no significant differences in innovation levels between treatment and control groups before M&As, thus satisfying the parallel trends assumption. Besides, post-M&A coefficients show significant positive effects, particularly in the first three years after M&A, demonstrating that M&As had a substantial and lasting positive impact on SOEs’ innovation capabilities. The coefficients’ values and significance levels show a temporary decline starting at *T*_+5_, possibly due to market fluctuations during China’s economic transition period.

**Table 5:** Dynamic effects of M&A on innovation outputs.

	(1)	(2)	(3)	(4)
	*Patent apply*		*Patent_inv apply*	
*M&A *× *T*_*-3*_	0.059	(0.048)	0.049	(0.041)
*M&A *× *T*_*-2*_	0.061	(0.054)	0.026	(0.046)
*M&A *× *T*_* + 0*_	0.211***	(0.064)	0.117**	(0.055)
*M&A *× *T*_* + 1*_	0.335***	(0.078)	0.241***	(0.070)
*M&A *× *T*_* + 2*_	0.345***	(0.088)	0.202***	(0.078)
*M&A *× *T*_* + 3*_	0.276***	(0.100)	0.248***	(0.086)
*M&A *× *T*_* + 4*_	0.394***	(0.111)	0.292***	(0.098)
*M&A *× *T*_* + 5*_	0.351***	(0.120)	0.256**	(0.106)
*M&A *× *T*_* + 6*_	0.248*	(0.128)	0.221**	(0.114)
*M&A *× *T*_* + 7*_	0.353***	(0.135)	0.276**	(0.121)
*M&A *× *T*_* + 8*_	0.396***	(0.148)	0.281**	(0.136)
*M&A *× *T*_* + 9*_	0.313***	(0.177)	0.283*	(0.154)
Constant	2.183*	(0.019)	1.525***	(0.017)
Firm and year-fixed effects	Yes		Yes	
Observations	9,695		9,695	
Adjusted R^2^	0.8040		0.7936	

*Table notes* This table presents the dynamic effects of M&As on innovation outputs by examining pre- and post-M&A periods from *T*_*-3*_ to *T*_*+9*_, where *T* represents the M&A year. Columns (1)-(2) and (3)-(4) report effects on total patent applications and invention patent applications, respectively. The analysis uses OLS regression with robust standard errors clustered at firm level. *T*_*-1*_ serves as the reference period. Significance levels: *** p < 0.01, ** p < 0.05, * p < 0.1.

#### 5.4.2. Placebo tests.

**Fig 3:**
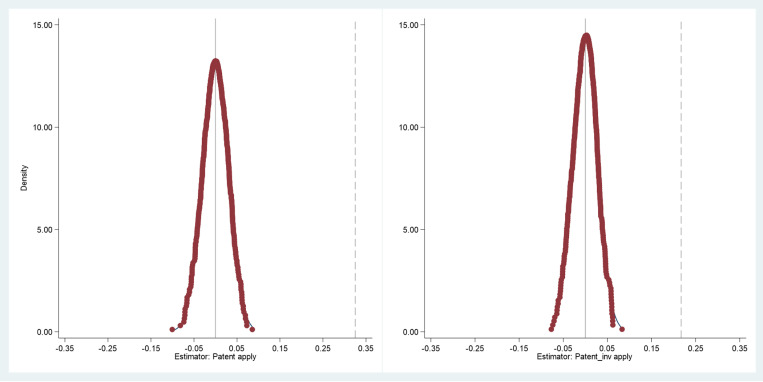
Distribution of Placebo Test coefficients. This figure presents the kernel density distribution of coefficients from 500 placebo tests where treatment status is randomly assigned. The dashed vertical line indicates our baseline regression coefficient (0.313). The significant deviation of our actual estimate from the placebo distribution supports the robustness of our findings. The horizontal axis represents coefficient values and the vertical axis represents density.

To address endogeneity concerns and validate the reliability of our findings, we conducted placebo tests. Specifically, this approach examines whether our findings are merely due to chance or other unobserved factors by randomly assigning treatment groups. We performed 500 iterations and compared the results with baseline estimates. The results are presented in Fig 3.

The test results reveal that the baseline regression coefficients exhibit distinct distribution patterns under random sampling, significantly deviating from the main distribution interval of placebo estimates. The kernel density distribution of placebo test coefficients clusters tightly around zero, displaying normal distribution characteristics. This distribution pattern strongly indicates that our main findings are not due to random factors or unobserved variables. The fact that baseline regression coefficients fall in the distribution tail further supports the credibility of our policy effect estimates. Additionally, the concentration of kernel density distribution reflects the stability of our econometric model.

#### 5.4.3. Granger causality tests.

To further address potential endogeneity concerns, particularly the reverse causality issue that may persist even after PSM-DID and placebo tests, we employed Granger causality tests. These tests provide an examination of the potential reverse causality between M&As and innovation in our matched sample.

Our Granger causality analysis employed multiple technical indicators, including p-values to assess the significance of unidirectional causality and model fit indicators (AIC and BIC). The results in Table 6 demonstrate no significant causal relationships between innovation activities and M&A decisions across different lag periods (2, 4, and 6 periods), for both total patent applications and invention patent applications.

**Table 6:** Granger causality test results.

Dependent variable	Independent variable	Lag period	P-value	AIC	BIC
*Patent apply*	*M&A×Post*	2	0.351	4.674	6.459
*M&A × Post*	*Patent apply*	2	0.956	4.674	6.459
*Patent apply*	*M&A × Post*	4	0.334	5.465	7.778
*M&A × Post*	*Patent apply*	4	0.970	5.465	7.778
*Patent apply*	*M&A × Post*	6	0.948	6.621	9.846
*M&A × Post*	*Patent apply*	6	0.511	6.621	9.846
*Patent_inv apply*	*M&A × Post*	2	0.090	1.857	3.625
*M&A × Post*	*Patent_inv apply*	2	0.998	1.857	3.625
*Patent_inv apply*	*M&A × Post*	4	0.036	2.119	4.406
*M&A × Post*	*Patent_inv apply*	4	0.901	2.119	4.406
*Patent_inv apply*	*M&A × Post*	6	0.000	2.802	5.994
*M&A × Post*	*Patent_inv apply*	6	0.215	2.802	5.994

*Table notes* This table presents Granger causality test results examining the causal relationship between M&As and innovation measures. The upper panel shows results for total patent applications, while the lower panel shows results for invention patent applications. For each innovation measure, we test both directions of causality with different lag periods (2, 4, and 6 periods). A significant p-value when *M&A *× *Post* is the independent variable, combined with an insignificant p-value when the patent measure is the independent variable, suggests that M&As Granger-cause innovation but not vice versa. AIC (Akaike Information Criterion) and BIC (Bayesian Information Criterion) are model fit statistics.

Conversely, the test results revealed that M&As significantly enhance firms’ invention innovation, with statistical significance at 5% and 1% levels for 4-period and 6-period lags, respectively. The absence of significant effects in the 2-period lag aligns with the inherent nature of innovation processes, which require substantial time for resource integration and strategy adjustment. This temporal pattern reflects the gradual implementation of organizational changes. These findings support our core argument that M&As drive innovation enhancement rather than the reverse.

#### 5.4.4. Supplementary control variables.

To systematically address potential omitted variable concerns, we focused on two key aspects that could influence our results: firm-level unobservable characteristics (such as management capabilities and organizational culture) and industry-level dynamics (such as industrial policies and market competition). To further enhance our analysis, we conducted robustness tests by including several supplementary control variables including employee size (the natural logarithm of employee), intangible assets ratio, investment efficiency (Richard, 2006), and industry competition (Lerner index). In Table 7, The positive effect of M&As on patent applications remains robust and statistically significant at the 1% level, with coefficients of 0.341 and 0.346. Furthermore, the moderating effect of mixed-ownership maintains its significance, as evidenced by the triple interaction terms (*Mixed-ownership *× *M&A *× *Post*) showing coefficients of 0.353 and 0.245.

**Table 7:** Analysis of supplementary control variables. The Impact of M&As on SOE Innovation with Additional Controls.

	(1)	(2)	(3)	(4)
	*Patent apply*	*Patent_inv apply*	*Patent apply*	*Patent_inv apply*
****M&A×Post****	0.341***	0.346***	0.222**	0.257***
	**(0.076)**	**(0.076)**	(0.086)	(0.083)
****Mixed-ownership × M&A × Post****			0.353***	0.245*
			(0.117)	**(0.127)**
Additional controls	Yes	Yes	Yes	Yes
Controls	Yes	Yes	Yes	Yes
Constant	0.997	1.505**	1.019	1.486**
	(0.773)	(0.730)	(0.763)	(0.724)
Observations	6,248	5,490	6,248	5,490
Adjusted R^2^	0.572	0.596	0.574	0.596

*Table notes* This table reports PSM-DID regression results examining the robustness of M&As’ impact on innovation. Columns (1) and (2) report the baseline effects of M&As, while Columns (3) and (4) examine the differential effects of mixed-ownership M&As. All specifications include firm and year fixed effects, baseline controls (defined in Table 1), and additional controls. The additional controls include: (1) employee size, measured as the natural logarithm of total employees; (2) intangible assets ratio, calculated as intangible assets divided by total assets; (3) investment efficiency, calculated from the model of Richard [103]; (4) industry competition, measured by the Lerner index calculated as the weighted average of firms’ operating profit margins, where the weights are the ratios of firm sales to industry total sales. Robust standard errors are clustered at the firm level and shown in parentheses. Significance levels: *** p < 0.01, ** p < 0.05, * p < 0.1.

While these empirical results provide support for our baseline findings, we remain mindful of potential limitations in fully capturing all relevant factors in our analysis.

#### 5.4.5. Alternative dependent variables.

To verify the robustness of our findings and address potential measurement bias, we employ three alternative measures of innovation outputs. First, we use the natural logarithm of granted patents (*Patent granted*) instead of patent applications. This addresses potential concerns about using patent applications alone, as granted patents represent validated technological innovations. The results in Table 8 further support our main conclusions. The patent grant analysis shows that M&A activities significantly promote the growth of patent grants, with this effect being more pronounced in mixed-ownership M&As. Specifically in Table 8, the coefficient for patent grants after M&As is 0.354 (p < 0.01), while for mixed-ownership M&As, it reaches 0.415 (p < 0.01).

**Table 8:** Analysis of alternative dependent variable. Impact of M&As on SOE Patent Authorizations.

	(1)	(2)	(3)	(4)
	*Patent granted*	*Patent_inv granted*	*Patent granted*	*Patent_inv granted*
***M&A×Post***	0.354***	0.259***	0.208**	0.143
	(0.079)	(0.083)	(0.089)	(0.098)
***Mixed-ownership × M&A × Post***			0.415***	0.297**
			(0.140)	(0.132)
Controls	Yes	Yes	Yes	Yes
Constant	0.863	1.445**	0.798	1.404**
	(0.678)	(0.639)	(0.673)	(0.640)
Observations	5,360	4,026	5,360	4,026
Adjusted R^2^	0.564	0.573	0.566	0.573

*Table notes* This table presents regression results using patent authorizations (granted patents) as alternative innovation measures to test the robustness of our findings. Columns (1) and (2) report the baseline effects of M&As on total granted patents and granted invention patents, respectively. Columns (3) and (4) examine the differential effects between mixed-ownership M&As and SOE-to-SOE M&As. The analysis uses PSM-DID method with firm and year fixed effects. Control variables are consistent with the baseline regression in Table 4. Robust standard errors are clustered at the firm level and shown in parentheses. Significance levels: *** p < 0.01, ** p < 0.05, * p < 0.1.

Second, following Mao and Zhang [90], we examine patent citations as a measure of innovation quality in Table 9. To address citation truncation issues [91], we use both raw citation counts (natural logarithm of citations, *Citation*1) and industry-standardized citations (*Citation*2, calculated by subtracting the industry mean from citations and dividing by the industry mean). Mixed-ownership M&As demonstrate significantly positive effects on both raw citations (0.232, p < 0.1) and industry-standardized citations (0.814, p < 0.05).

**Table 9:** Analysis of alternative dependent variable. Impact of M&As on SOE Patent Citations.

	(1)	(2)	(3)	(4)
	*Citation*1	*Citation*2	*Citation*1	*Citation*2
***M&A×Post***	0.010	0.581**	-0.074	0.299
	(0.103)	(0.256)	(0.109)	(0.290)
***Mixed-ownership × M&A × Post***			0.232*	0.814**
			**(0.137)**	(0.327)
Controls	Yes	Yes	Yes	Yes
Constant	1.601	-3.904**	1.561	1.404**
	(0.974)	(1.829)	(0.974)	(0.640)
Observations	6,728	9,686	6,728	9,686
Adjusted R^2^	0.621	0.435	0.622	0.437

Table notes This table presents regression results using patent citations as alternative innovation measures to test the robustness of our findings. Columns (1) and (2) report the baseline effects of M&As on patent citations (*Citation*1) and patent citations standardized by industry average (*Citation*2), respectively. Columns (3) and (4) examine the differential effects between mixed-ownership M&As and SOE-to-SOE M&As. The analysis uses PSM-DID method with firm and year fixed effects. Control variables are consistent with the baseline regression in Table 4. Robust standard errors are clustered at the firm level and shown in parentheses. Significance levels: *** p < 0.01, ** p < 0.05, * p < 0.1.

Third, following Akcigit et al. [92], we use patent breadth as a proxy for innovation quality. Patent breadth is measured using the Herfindahl-Hirschman Index at the main group level of International Patent Classification (IPC) classification codes assigned by China National Intellectual Property Administration (CNIPA), where patents can have multiple classification codes. A higher index indicates broader and more complex technological knowledge coverage. We calculate the average breadth for both patent applications and granted patents each year. Table 10 shows that M&As enhance patent breadth, with mixed-ownership M&As showing additional positive effects on both application breadth (0.069, p < 0.05) and granted patent breadth (0.078, p < 0.05). These consistent findings across different innovation measures suggest that mixed-ownership reform through M&As enhances both the quantity and quality of SOE innovation.

**Table 10:** Analysis of alternative dependent variable. Impact of M&As on SOE Patent Breadth.

	(1)	(2)	(3)	(4)
	*Breadth apply*	*Breadth granted*	*Breadth apply*	*Breadth granted*
***M&A×Post***	0.092***	0.093***	0.068***	0.066***
	(0.020)	(0.022)	(0.023)	(0.024)
***Mixed-ownership × M&A × Post***			0.069**	0.078**
			**(0.031)**	(0.032)
Controls	Yes	Yes	Yes	Yes
Constant	0.733***	0.603***	0.715***	0.587***
	(0.174)	(0.182)	(0.173)	(0.182)
Observations	9,731	9,716	9,731	9,716
Adjusted R^2^	0.408	0.415	0.409	0.416

*Table notes* This table presents regression results using patent breadth as alternative innovation measures to test the robustness of our findings. Columns (1) and (2) report the baseline effects of M&As on the breadth of patent applications and patent authorizations, respectively. Columns (3) and (4) examine the differential effects between mixed-ownership M&As and SOE-to-SOE M&As. The analysis uses PSM-DID method with firm and year fixed effects. Control variables are consistent with the baseline regression in Table 4. Robust standard errors are clustered at the firm level and shown in parentheses. Significance levels: *** p < 0.01, ** p < 0.05, * p < 0.1.

#### 5.4.6. Alternative regression models.

In our baseline analysis, we employ truncated OLS regression focusing on firms with existing patent applications, given the high frequency of zero observations in patent data. To complement this baseline approach, Table 11 presents two additional robustness tests.

**Table 11:** Analysis of alternative regression models. M&A and SOE Innovation.

	(1)	(2)	(3)	(4)	(5)	(6)	(7)	(8)
	Full sample OLS				Tobit			
	*Patent apply*	*Patent_inv apply*	*Patent apply*	*Patent_inv apply*	*Patent apply*	*Patent_inv apply*	*Patent apply*	*Patent_inv apply*
***M&A × Post***	0.442***	0.450***	0.375***	0.369***	0.442***	0.450***	0.375***	0.369***
	(0.073)	(0.073)	(0.088)	(0.080)	(0.071)	(0.071)	(0.085)	(0.078)
***Mixed-ownership × M&A × Post***			0.195*	0.232*			0.195*	0.232*
			(0.117)	(0.123)			(0.114)	(0.120)
Controls	Yes	Yes	Yes	Yes	Yes	Yes	Yes	Yes
Constant	1.930***	1.250*	1.939***	1.213*	0.455	-0.981*	0.491	-0.978*
	(0.672)	(0.638)	(0.670)	(0.638)	(0.800)	(0.534)	(0.785)	(0.531)
Observations	9,613	9,701	9,613	9,701	9,613	9,701	9,613	9,701
Adjusted R^2^	0.601	0.565	0.601	0.565	0.235	0.231	0.235	0.231

*Table notes* This table presents robustness test results using alternative regression models. Columns (1)-(4) report full sample OLS regression results including observations with zero patent applications, while Columns (5)-(8) report Tobit regression results addressing the left-censoring of patent data at zero. For each model specification, we examine both the baseline effects of M&As (Columns 1–2 and 5–6) and the differential effects of mixed-ownership M&As (Columns 3–4 and 7–8). All regressions include firm and year fixed effects and the same control variables as in Table 4. Robust standard errors are clustered at the firm level and shown in parentheses. Significance levels: *** p < 0.01, ** p < 0.05, * p < 0.1.

First, we extend our analysis to include the full sample through OLS regression, incorporating firms with zero patent applications. The results show that M&A events (*M&A* × *Post*) maintain their significant positive effect on innovation outputs even in this broader sample. Second, considering the non-negative nature of patent counts, we employ Tobit models to address potential left-censoring issues. This alternative specification also supports our main findings.

Across both complementary approaches, the interaction terms (*Mixed-ownership* × *M&A* × *Post*) remain positive and statistically significant at the 10% level, reinforcing our conclusions about the positive moderating effect of mixed-ownership in M&As on SOE innovation.

#### 5.4.7. Alternative explanations and subsample analysis.

To address potential omitted variable bias, this section conducts subsample analyses focusing on two key dimensions that could confound our results: payment methods and industry characteristics. These analyses systematically rule out alternative explanations and strengthen the reliability of our findings.

First, we investigate whether our results are driven by changes in ownership structure through payment methods. By excluding M&A cases with equity payment, we can isolate the pure effect of M&As from ownership changes. Columns (1)-(4) of Table 12 reveal that M&As maintain their significant positive effect on innovation (coefficients: 0.293 and 0.323, p < 0.01), confirming that the innovation enhancement is not merely due to ownership structure changes through equity payments.

**Table 12:** Impact of M&As on SOE technological innovation. Subsample Analysis Based on Payment Methods and Industry Characteristics.

	(1)	(2)	(3)	(4)	(5)	(6)	(7)	(8)
	Excluding Equity-Paid M&As				Excluding Public Welfare Industries			
	*Patent apply*	*Patent_inv apply*	*Patent apply*	*Patent_inv apply*	*Patent apply*	*Patent_inv apply*	*Patent apply*	*Patent_inv apply*
***M&A × Post***	0.293***	0.323***	0.165*	0.224**	0.318***	0.326***	0.210**	0.247***
	(0.080)	(0.081)	(0.093)	(0.087)	(0.079)	(0.076)	(0.093)	(0.086)
***Mixed-ownership × M&A × Post***			0.366***	0.276**			0.302**	0.212*
			(0.122)	(0.139)			(0.120)	(0.121)
Controls	Yes	Yes	Yes	Yes	Yes	Yes	Yes	Yes
Constant	1.784***	2.075**	1.785***	2.050**	0.994	1.686**	0.999	1.685**
	(0.679)	(0.825)	(0.671)	(0.820)	(0.694)	(0.684)	(0.690)	(0.672)
Observations	5,846	5,153	5,846	5,153	5,899	5,173	5,899	5,173
Adjusted R^2^	0.560	0.578	0.562	0.579	0.591	0.615	0.592	0.616

*Table notes* This table reports subsample regression results examining the robustness of our findings. Columns (1)-(4) present results after excluding equity-paid M&As to isolate the effects of cash-only transactions. Columns (5)-(8) present results after excluding public welfare industries (utilities, transportation, and environmental protection) to focus on market-oriented sectors. For each subsample, we examine both the baseline effects of M&As (Columns 1–2 and 5–6) and the differential effects of mixed-ownership M&As (Columns 3–4 and 7–8). All regressions include firm and year fixed effects and the same control variables as in Table 4. Robust standard errors are clustered at the firm level and shown in parentheses. Significance levels: * p < 0.01, p < 0.05, * p < 0.1.

Second, we address industry-specific confounding factors by excluding monopolistic industries and public service SOEs, which may have distinct innovation patterns and reform priorities. Results in columns (5)-(8) of Table 12 show that M&As continue to significantly boost innovation (coefficients: 0.318 and 0.326, p < 0.01) with comparable magnitudes to the full sample, demonstrating that our findings are not driven by industry-specific characteristics.

Importantly, the interaction term *Mixed-ownership* × *M&A* × *Post* maintains its significant positive coefficient across all subsamples. This consistent pattern provides robust evidence that mixed-ownership reform enhances the innovation-promoting effect of M&As through improved governance mechanisms, ruling out alternative explanations related to payment methods or industry characteristics.

### 5.5. Heterogeneity analysis

In this section, we focused on *Patent apply* to examine how transaction characteristics, SOEs’ investment and production conditions and the external institutional environment explain the stronger positive impact on technological innovation in mixed-ownership M&As.

#### 5.5.1. Control rights transfer and transaction size.

This section examines how M&A depth and scale affect innovation outputs. We conduct heterogeneity analysis through two dimensions - control rights transfer and transaction size - to deepen our understanding of mixed-ownership M&A mechanisms. Our findings reveal that control rights transfer significantly influences the relationship between target firms’ ownership nature and technological innovation outputs. Specifically, as shown in columns (1)-(2) of Table 13, mixed-ownership M&As involving control rights transfer demonstrate more significant positive effects on technological innovation (coefficient difference significant at 5% level), supporting our theoretical analysis of integrating diverse innovation strategies and management systems.

**Table 13:** Impact of mixed-ownership M&As on SOE technological innovation. Heterogeneity Analysis by Transaction Characteristics.

	(1)	(2)	(3)	(4)
	Control unchanged	Control transfer	Small volume	Large volume
***M&A × Post***	0.292***	0.055	0.229**	0.209*
	(0.101)	(0.135)	(0.114)	(0.121)
***Mixed-ownership × M&A × Post***	0.119	0.554***	0.278*	0.375**
	(0.143)	(0.173)	(0.157)	(0.173)
Controls	Yes	Yes	Yes	Yes
Constant	1.788**	2.249**	1.769**	2.544**
	(0.812)	(0.958)	(0.828)	(0.984)
Observations	4,315	2,636	3,692	3,226
Adjusted R^2^	0.530	0.607	0.574	0.561
Difference in coefficients	-0.435** **(p-value = 0.022)**		-0.097 (p-value = 0.451)	

*Table notes* This table presents heterogeneity analysis results examining how M&As’ effects vary with transaction characteristics. Columns (1)-(2) compare cases where control rights remain unchanged versus transfer to the acquirer. Columns (3)-(4) compare transactions based on deal size (small versus large, split at the median). The dependent variable is total patent applications. All models include firm and year fixed effects and the same control variables as in Table 4. Robust standard errors are clustered at the firm level and shown in parentheses. The bottom panel reports differences in *Mixed-ownership* × *M&A *× *Post* coefficients between paired subsamples (control unchanged vs. transfer; small vs. large volume), with p-values in parentheses. Significance levels: *** p < 0.01, ** p < 0.05, * p < 0.1.

Notably, in M&As without control rights transfer (column 2 of Table 13), we observe non-significant interaction terms (*Mixed-ownership* × *M&A* × *Post*). This suggests that without deep technical and managerial integration, SOEs cannot fully leverage their innovation advantages when acquiring private enterprises. The lack of substantive integration may lead to the continued coexistence of different organizational cultures, hindering innovation synergy; differences in incentive mechanisms between SOEs and private enterprises may persist without deep integration, affecting innovation motivation; without control transfer, resources may not flow and optimize effectively, limiting innovation potential. This non-significant result emphasizes the importance of deep integration for achieving innovation synergy, particularly in the context of significant cultural differences between Chinese SOEs and private enterprises.

The specific manifestations of deep integration include several aspects: First, at the innovation strategy level, systematic integration planning of R&D directions, technological roadmaps, and innovation resources of both parties is required. Second, at the management system level, a unified innovation project evaluation and resource allocation mechanism should be established to ensure efficient distribution of innovation resources. Third, at the talent management level, effective incentive mechanisms need to be designed to promote technical talent exchange and cooperation. Finally, at the corporate culture level, efforts should be made to build an open innovation organizational atmosphere and eliminate integration barriers caused by cultural differences between state-owned and private enterprises. The implementation of these deep integration measures is crucial for achieving innovation synergy after mixed-ownership M&As.

On the other hand, columns (3)-(4) of Table 13 indicate that as long as M&As promote substantial integration between SOEs and private enterprises, transaction size does not significantly affect the innovation-promoting effect of mixed-ownership M&As (*Mixed-ownership* × *M&A* × *Post* coefficients are both significantly positive, with no significant difference between coefficients). This result emphasizes the importance of substantive integration for innovation outputs, rather than merely depending on transaction size.

These findings have implications for regulators, corporate management, and policymakers. When approving and implementing mixed-ownership M&As, more attention should be paid to whether transactions generate subsequent substantive integration plans, rather than just focusing on transaction size. Corporate management should develop detailed integration plans when conducting mixed-ownership M&As, especially regarding innovation strategy, management systems, and corporate culture. Policymakers might consider developing policies supporting deep integration in mixed-ownership enterprises, such as tax incentives or innovation subsidies, to promote more effective innovation synergy.

#### 5.5.2. R&D investment and production efficiency.

Based on open innovation theory, we examine how SOEs’ absorptive capacity and complementary capabilities affect mixed-ownership M&As’ innovation outcomes. This section explores the moderating effects of R&D intensity (R&D expenditure/ operating revenue) and total factor productivity (TFP calculated using the OP model) on innovation performance after mixed-ownership M&As.

Our findings in Table 14 show that SOEs’ prior R&D investment significantly moderates mixed-ownership M&As’ innovation effects. For low R&D investment SOEs, the coefficient of *Mixed-ownership* × *M&A* × *Post* (0.227) is not statistically significant. This suggests that insufficient R&D foundation limits firms’ ability to absorb new knowledge and integrate cultures during post-M&A integration, thereby challenging traditional assumptions about automatic innovation gains from M&As. In contrast, high R&D investment SOEs show a significant positive effect (coefficient = 0.328, p < 0.05), highlighting how existing innovation foundations enhance resource complementarity and knowledge spillovers.

**Table 14:** Impact of mixed-ownership M&As on SOE technological innovation. Heterogeneity analysis by firm characteristics.

	(1)	(2)	(3)	(4)
	Low *R&D*	High *R&D*	Low *TFP*	High *TFP*
***M&A × Post***	0.243**	0.017	0.153	0.232*
	(0.112)	(0.110)	(0.104)	(0.120)
***Mixed-ownership × M&A × Post***	0.227	0.328**	0.360**	0.047
	(0.152)	(0.158)	(0.146)	(0.157)
Controls	Yes	Yes	Yes	Yes
Constant	1.243	2.265**	1.345	1.856*
	(0.790)	(0.959)	(1.154)	(1.025)
Observations	4,151	2,685	3,026	3,838
Adjusted R^2^	0.551	0.685	0.531	0.633
Difference in coefficients	-0.101 (p-value = 0.342)		0.313*** **(p-value = 0.000)**	

*Table notes* This table presents heterogeneity analysis results examining how M&As’ effects vary with firm characteristics. Columns (1)-(2) compare firms with low versus high R&D investment intensity (split at the median). Columns (3)-(4) compare firms with low versus high total factor productivity (TFP, split at the median). The dependent variable is total patent applications. All models include firm and year fixed effects and the same control variables as in Table 4. Robust standard errors are clustered at the firm level and shown in parentheses. The bottom panel reports differences in *Mixed-ownership* × *M&A *× *Post* coefficients between paired subsamples (low vs. high R&D; low vs. high TFP), with p-values in parentheses. Significance levels: *** p < 0.01, ** p < 0.05, * p < 0.1.

Regarding efficiency, less efficient SOEs benifit more from mixed-ownership M&As. As shown in columns (3) and (4) of Table 14, the low-efficiency sample shows a significant positive effect (coefficient = 0.360, p < 0.05), while the high-efficiency SOEs sample show no significant effect (coefficient = 0.047). The coefficient difference in is significant at the 1% level. This suggests that low-efficiency SOEs gain more from market-oriented reforms through improved management and governance, while already-efficient SOEs see limited additional benefits.

In conclusion, mixed-ownership M&As most benefit SOEs with strong R&D foundations but lower operational efficiency. These findings not only deepen our understanding of how mixed-ownership M&As promote SOE innovation but also provide important guidance for policymakers and managers in selecting suitable SOE candidates for mixed-ownership reform, emphasizing the importance of existing innovation foundation and operational characteristics.

#### 5.5.3. Regional institutional environment.

This section uses the marketization index of Chinese provinces from the National Economic Research Institute (NERI) to examine how institutional environment influences innovation outputs following mixed-ownership M&As. We focus on two key dimensions: the government-market relationship index (which evaluates the government’s role in resource allocation, market intervention level, and government size) and the legal institutional environment index (which assesses the development of market intermediaries, rule of law compliance, and intellectual property protection). Higher indices indicate greater market development in these regions.

Our empirical analysis in Table 15 reveals that the institutional environment significantly influences the innovation effects of mixed-ownership M&As. In regions with high government intervention, mixed-ownership M&As demonstrate significant innovation advantages, with a regression coefficient of *Mixed-ownership* × *M&A* × *Post* 0.379 (p < 0.05). In contrast, this advantage becomes statistically insignificant (coefficient = -0.051) in more market-oriented environments. Similarly, mixed-ownership M&As show stronger innovation advantages in regions with weaker legal frameworks (coefficient = 0.490, p < 0.01), while this advantage disappears (coefficient = 0.020) in regions with well-developed legal systems.

**Table 15:** Impact of mixed-ownership M&As on SOE technological innovation. Heterogeneity analysis by institutional environment.

	(1)	(2)	(3)	(4)
	Government-market relationship		Intermediary organizations and legal systems	
	Intervention	Marketization	Developing	Well-developed
***M&A×Post***	-0.020	0.528***	-0.085	0.521***
	(0.130)	(0.109)	(0.124)	(0.124)
***Mixed-ownership × M&A × Post***	0.379**	-0.051	0.490***	0.020
	(0.164)	(0.144)	(0.156)	(0.163)
Controls	Yes	Yes	Yes	Yes
Constant	1.923*	3.283***	0.493	3.786***
	(1.077)	(0.888)	(1.006)	(1.037)
Observations	3,001	3,899	3,587	3,323
Adjusted R^2^	0.666	0.592	0.601	0.676
Difference in coefficients	0.430* **(p-value = 0.088)**		0.470* **(p-value = 0.079)**	

*Table notes* This table presents heterogeneity analysis results examining how M&As’ effects vary with institutional environments. Columns (1)-(2) compare regions with high government intervention versus high marketization based on the government-market relationship index. Columns (3)-(4) compare regions with developing versus well-developed intermediary organizations and legal systems based on the institutional development index. The dependent variable is total patent applications. All models include firm and year fixed effects and the same control variables as in Table 4. Robust standard errors are clustered at the firm level and shown in parentheses. The bottom panel reports differences in *Mixed-ownership* × *M&A *× *Post* coefficients between paired subsamples (intervention vs. marketization; developing vs. well-developed), with p-values in parentheses. Significance levels: *** p < 0.01, ** p < 0.05, * p < 0.1.

These findings reveal an important mechanism: as government intervention decreases and market mechanisms improve, the differences between state-owned and private enterprises in resource acquisition and management philosophy gradually narrow. Specifically, in more market-oriented environments, SOEs face harder budget constraints and stronger market competition, forcing them to adopt more market-oriented management practices and innovation strategies. Meanwhile, private enterprises gain better access to resources through mature market mechanisms. This convergence in operational practices and resource accessibility weakens the impact of ownership type on post-M&A innovation.

In well-developed legal environments, both state-owned and private enterprises can accumulate innovation resources through fair competition, as enhanced intellectual property protection reduces expropriation risks, standardized market intermediaries facilitate technology transactions, and improved contract enforcement ensures fair competition. These institutional safeguards enable enterprises of different ownership types to focus on market-based innovation strategies rather than relying on administrative advantages, further reducing the innovation gap between these two types of enterprises and diminishing the advantages of mixed-ownership M&As.

The empirical results from Table 15 further indicate that mixed-ownership M&As generate more significant positive innovation effects in regions with lower levels of marketization compared to more developed regions. This finding suggests that the integration of state and private capital may partially compensate for the adverse effects of insufficient market development. Particularly in less developed market environments, mixed-ownership M&As show better innovation enhancement effects compared to M&As between purely state-owned enterprises. These findings not only extend resource dependence theory and agency theory in different market environments but also provide implications for policymakers: mixed-ownership reform may be more necessary in regions with lower levels of marketization, though its advantages may gradually diminish as marketization levels increase. These results deepen our understanding of China’s regional development imbalances and provide theoretical support for developing differentiated reform strategies.

These findings have important policy implications for regions with different levels of market development. For less marketized regions, priorities should focus on streamlining mixed-ownership M&A frameworks to reduce costs. For more marketized regions, focus should shift to developing market-oriented innovation evaluation systems. This differentiated policy design will help regions more effectively advance mixed-ownership reform according to their development stages.

While our current analysis captures the broad institutional framework through marketization indices, these micro-level dynamics warrant further investigation to fully understand the complex interplay between institutional environments and innovation outputs in China’s mixed-ownership reforms.

### 5.6. Mechanism analysis: non-state shareholder participation

This section explores how mixed-ownership M&As enhance innovation through non-state shareholders’ participation in corporate governance. Drawing on institutional theory and resource dependence theory, we examine how different M&As types affect this participation. Mixed-ownership M&As optimize the institutional environment through market mechanisms, while the integration of state and private resources can create unique innovation advantages. We measure non-state shareholder participation using the *Non-state governance* indicator - the proportion of directors, executives, and supervisors appointed by non-state shareholders - reflecting their influence on corporate decision-making.

The empirical analysis reveals distinct effects between mixed-ownership and SOE-to-SOE M&As on non-state shareholder participation. As shown in Table 16, while general M&As (*M&A* × *Post*) show no significant impact on non-state governance (coefficient = 0.490, p > 0.10), mixed-ownership M&As demonstrate a substantial positive effect (coefficient = 0.971, p < 0.05). This effect notably exceeds that of SOE-to-SOE M&As (coefficient = 0.581), with mixed-ownership M&As showing stronger effectiveness (coefficient = 1.098) in promoting non-state participation and fundamental governance transformation.

**Table 16:** Impact of M&As on non-state shareholder participation in governance: mechanism analysis.

	(1)	(2)	(3)
	Full sample	*Mixed-ownership* = 0	*Mixed-ownership* = 1
***M&A×Post***	0.490	0.581*	1.098**
	(0.338)	(0.337)	(0.457)
***Mixed-ownership × M&A × Post***	0.971**		
	(0.464)		
Controls	Yes	Yes	Yes
Constant	8.074***	6.175*	11.648**
	(3.111)	(3.235)	(5.784)
Observations	8,327	5,759	2,568
Adjusted R^2^	0.367	0.374	0.370
Difference in coefficients		-0.517** **(p-value = 0.030)**	

*Table notes* This table presents mechanism analysis results examining how M&As affect non-state shareholder participation in governance, measured as the ratio of directors, executives, and supervisors appointed by non-state shareholders to total board members and executives. Column (1) reports results for the full sample. Columns (2) and (3) present subsample analysis comparing SOE-to-SOE M&As (*Mixed-ownership* = 0) with mixed-ownership M&As (*Mixed-ownership* = 1). All models include firm and year fixed effects and the same control variables as in Table 4. Robust standard errors are clustered at the firm level and shown in parentheses. The bottom panel reports the difference in *M&A* × *Post* coefficients between subsamples (*Mixed-ownership* = 1 vs. 0), with p-value in parentheses. Significance levels: *** p < 0.01, ** p < 0.05, * p < 0.1.

Enhanced non-state participation improves innovation through several mechanisms: introducing market-oriented management and decision-making, enabling more effective resource allocation toward high-potential projects and fostering innovation through diverse in governance perspectives.

These findings support our third hypothesis (H3): non-state shareholder participation is a mechanism through which mixed-ownership M&As promote innovation. This extends resource dependence theory in emerging market by showing how governance diversification improves both resource complementarity and utilization efficiency. It also advances agency theory by demonstrating how mixed-ownership M&As create innovation incentives through non-state shareholder participation, highlighting the importance of translating ownership structure diversification into substantive governance engagement.

## 6. Conclusion and discussion

### 6.1. Key findings

This study demonstrates how mixed-ownership acquisitions active non-state shareholders participation in corporate governance, thereby significantly enhancing SOE innovation. output through active participation of non-state shareholders in corporate governance. These findings not only extend the existing literature on mixed-ownership economic consequences but also provide new theoretical insights into the relationship between acquisition types and innovation outputs. While this may seem to contradict studies that view SOEs negatively based on their political identity, this apparent contradiction can be understood through the complementary nature of this partnership reveals a unique synergy: SOEs contribute through risk tolerance and long-term stability, while private partners bring market agility and operational efficiency. These findings extend current literature on mixed-ownership effects while offering fresh theoretical perspectives on how acquisition types influence innovation outcomes.

Our heterogeneity analysis uncovers three critical boundary conditions. First, innovation gains are most pronounced in transactions involving control rights transfer, and in SOEs with strong innovation resources but low efficiency. Second, the positive effects are stronger in regions with lower market development, though these benefits tend to decrease as markets mature. Third, SOEs without adequate R&D foundations show limited innovation improvements from mixed-ownership acquisitions. These insights significantly contribute to institutional theory by illustrating how ownership hybridity can effectively address the “innovation paradox” commonly observed in transitional economies. Furthermore, our findings on control rights transfer complement resource dependence theory, emphasizing the importance of deep integration mechanisms for achieving innovation synergies.

### 6.2. Beyond the Chinese context

Our institutional analysis reveals several significant distinctions in mixed-ownership approaches across different national contexts. First, regarding market maturity effects, while the Chinese context demonstrates pronounced efficiency gains in less developed markets (due to greater potential for improvement), mature economies typically rely on sophisticated public-private partnerships [27,93], as exemplified by the French National AI Laboratory’s collaborative initiatives with private sector entities [94]. These differences suggest that mixed-ownership reforms must adapt their focus and methods according to market development stages to optimize innovation outcomes.

Second, the role of government exhibits marked variation. Research shows that the degree of control rights transfer significantly correlates with innovation output. The Chinese approach favors direct equity participation [95,96], whereas non-Chinese models, particularly in developed economies, prefer indirect methods such as policy guidance and tax incentives, resulting in lower ownership intervention [97,98]. The US SBIR program illustrates this contrast through its emphasis on tax-leveraged research and development incentives rather than direct ownership intervention [99,100]. The varying depths of ownership intervention affect enterprises’ innovation resource allocation efficiency, decision-making mechanisms, and risk-taking capacity.

Third, innovation orientations differ substantially. The Chinese mixed-ownership model leverages state-owned enterprises’ scale advantages and private sector’s market efficiency to drive systematic innovation improvements. This differs from western approaches that emphasize venture capital-driven breakthrough innovations [101], as seen in Israel’s cybersecurity sector [102]. This contrast highlights how mixed-ownership reforms can create unique synergies between state resources and market mechanisms to achieve balanced innovation outcomes.

These institutional variations reveal important insights for mixed-ownership reforms. The Brazilian electricity sector reform offers a particularly instructive intermediate model that balances state control with market efficiency. In this model, while core infrastructure remains under state ownership to ensure strategic stability and public interest, private sector participation is actively encouraged in innovative service delivery. This creates a dynamic ecosystem where state-owned infrastructure serves as a foundation for market-driven innovation in service operations [49,52]. The Brazilian case demonstrates how maintaining strategic state control while introducing market mechanisms can effectively drive innovation - a valuable reference point for China’s ongoing mixed-ownership reforms.

### 6.3. Implications

Based on these findings, we propose several systematic policy recommendations to enhance innovation outcomes in mixed-ownership reforms. The regulatory framework should be recalibrated to emphasize post-merger integration mechanisms rather than traditional transaction metrics. Specifically, we advocate for the establishment of institutionalized knowledge transfer systems, exemplified by joint research and development centers that facilitate cross-ownership technological collaboration. These centers could be supported by targeted fiscal incentives, such as enhanced tax deductions for collaborative innovation outputs.

Furthermore, we recommend implementing dynamic governance mechanisms that align ownership structure adjustments with innovation performance metrics. Such mechanisms would create sustainable incentives for continuous innovation while maintaining appropriate checks and balances in corporate governance. The effectiveness of these mechanisms has been demonstrated in several successful mixed-ownership reforms in China’s telecommunications sector.

These recommendations require careful adaptation contextualized across different institutional contexts. In emerging economies like India and Brazil, emphasis should be placed on establishing patent co-management trusts to prevent technology misappropriation risks, particularly in regions with weaker institutional frameworks. This approach helps safeguard intellectual property while facilitating knowledge transfer. In developed markets like the EU and US, the focus shifts to antitrust compliance models, exemplified by successful partnerships between German “hidden champion” enterprises and state funds in forming Industry 4.0 standardization alliances. While ownership structures may vary across these contexts, the fundamental principles of protecting innovation assets and ensuring systematic integration remain essential for achieving optimal innovation outcomes.

### 6.4. Limitations

However, our study has several methodological limitations. First is the endogeneity issue: SOEs may strategically select private firms with high innovation potential as acquisition targets; firms’ existing innovation capabilities might influence their propensity to engage in mixed-ownership M&As; and time-varying unobservable factors could simultaneously affect both acquisition decisions and innovation outputs. While we attempt to address these issues through PSM-DID methodology and careful sample construction, certain challenges remain. Second, despite our comprehensive analytical approach, several unobservable factors could affect our results, including management capabilities, organizational culture, and dynamic local political environments. Additionally, the complex nature of China’s regional institutional environment poses challenges for controlling relevant factors.

Building on this, we suggest three directions for future research: methodologically, qualitative case studies could explore specific mechanisms through which mixed-ownership enhances innovation capabilities (Wang et al., 2021); theoretically, research could examine how institutional theory and resource dependence theory jointly explain mixed-ownership reform effects; contextually, studies could investigate how regional market development and industry characteristics influence the innovation outcomes of mixed-ownership M&As.

Finally, our findings have broad applicability. For government-controlled enterprises in other emerging economies, this study provides practical guidance on enhancing innovation capabilities through mixed ownership. For developed economies, despite different institutional contexts, the experience in integrating multiple resources and balancing regulation with market-oriented operations in public-private partnerships remains valuable. These cross-contextual applications demonstrate how ownership reform experiences can inform global governance innovations, deepening our understanding of the relationship between organizational innovation and ownership structure.

## Supporting information

S1 File Data. Raw data for mixed-ownership M&A and innovation output analysis.
(XLSX)
